# Stratifying Ovarian Cancer Risk Using Personal Health Data

**DOI:** 10.3389/fdata.2019.00024

**Published:** 2019-07-02

**Authors:** Gregory R. Hart, Bradley J. Nartowt, Wazir Muhammad, Ying Liang, Gloria S. Huang, Jun Deng

**Affiliations:** ^1^Department of Therapeutic Radiology, School of Medicine, Yale University, New Haven, CT, United States; ^2^Department of Obstetrics, Gynecology and Reproductive Sciences, School of Medicine, Yale University, New Haven, CT, United States

**Keywords:** ovarian cancer, neural network, risk stratification, personal health data, screening

## Abstract

**Purpose:** Screening the general population for ovarian cancer is not recommended by every major medical or public health organization because the harms from screening outweigh the benefit it provides. To improve ovarian cancer detection and survival many are looking at high-risk populations who would benefit from screening.

**Methods:** We train a neural network on readily available personal health data to predict and stratify ovarian cancer risk. We use two different datasets to train our network: The National Health Interview Survey and Prostate, Lung, Colorectal, and Ovarian Cancer Screening Trial.

**Results:** Our model has an area under the receiver operating characteristic curve of 0.71. We further demonstrate how the model could be used to stratify patients into different risk categories. A simple 3-tier scheme classifies 23.8% of those with cancer and 1.0% of those without as high-risk similar to genetic testing, and 1.1% of those with cancer and 24.4% of those without as low risk.

**Conclusion:** The developed neural network offers a cost-effective and non-invasive way to identify those who could benefit from targeted screening.

## Introduction

Ovarian cancer has a low incidence rate. However, ovarian cancer is the leading cause of death from gynecologic cancer and is the fifth most common cause of cancer death among US women (US Cancer Statistics Working Group, 2017). The high mortality rate is largely due to late stage diagnoses (Howlader et al., 2017). The 5-year relative survival rate for cancers diagnosed at a local stage is 93%, but 60% of women have distant spread of cancer at the time of diagnosis (Howlader et al., 2017). In early stages of ovarian cancer symptoms are often vague and non-specific (Goff et al., 2004).

Currently there are three methods commonly used for ovarian cancer detection. These are pelvic examination with bimanual palpation of the ovaries, transvaginal ultrasound, and testing serum for cancer antigen 125 (CA-125). However, none of these, when applied for screening the general population, reduce the mortality rate from ovarian cancer, which is why no major medical or public health organization recommends general screening for ovarian cancer (Brown et al., 2010; American Academy of Family Physicians, 2017; Committee on Gynecologic Practice, Society of Gynecologic Oncology, 2017; Grossman et al., 2018; Smith et al., 2018). The harms associated with false positives are non-negligible and result in an excess number of surgical procedures (Jacobs et al., 1999, 2015; Buys et al., 2011; Grossman et al., 2018). Therefore, there is ongoing research into improving serologic test and imaging, with the aim of minimizing false-positive results (Grossman et al., 2018).

Another approach to improving the performance of these screenings would be to identify those at high risk of developing ovarian cancer and to restrict screening to this subpopulation. Currently the high-risk population is identified based on the person's family history of breast, gynecologic, and colon cancer (Committee on Gynecologic Practice, Society of Gynecologic Oncology, 2017; Grossman et al., 2018). However, the majority of ovarian cancer diagnoses are not in women with first-degree relatives having ovarian or breast cancer. One study found only 18% of patients diagnosed with ovarian cancer to have inherited pathogenic mutations (Norquist et al., 2016). There have been several attempts to predict ovarian cancer risk, many of which rely on results of blood or genetic tests (Skates et al., 2003; Andersen et al., 2010; Pearce et al., 2013). Alternatively, two studies describe use of only readily available personal health data such as BMI, smoking habits, and age (Collins and Altman, 2013; Pfeiffer et al., 2013).

The goal of this work is to develop a predictive model that can identify a high-risk population who could benefit from screening, based on information that is routinely stored in electronic medical records (EMR), or easily obtainable from patients. However, rather than using traditional risk models we use a neural network (NN). Neural networks are a powerful non-linear statistical data modeling tool. They can capture interactions between various factors, allowing them to outperform standard statistical approaches, such as logistic regression, for complex systems like human health (Bishop, 2006). However, this power can also allow them to fit on noise and not generalize well. In particular this is a concern when the data comes from a single source, such as a specific brand of equipment (Kumar et al., 2012; Mackin et al., 2015). Accordingly we use two different datasets, the National Health Interview Survey (NHIS) (Blewett et al., 2016; Centers for Disease Control and Prevention, 2017) and Prostate, Lung, Colorectal, and Ovarian Cancer Screening Trial (PLCO) (Kramer et al., 1993; Buys et al., 2011), to train our model. Once the NN is trained and validated we show how it can be used to stratify patients in terms of ovarian cancer risk. Furthermore, we demonstrate our model's potential applications in identifying higher risk populations that could benefit from targeted screening.

## Methods

### Data Sources

In this study we used data from two different sources: the National Health Interview Survey (NHIS) (Blewett et al., 2016) and Prostate, Lung, Colorectal, and Ovarian Cancer Screening Trial (PLCO) (Buys et al., 2011). We trained our model on each dataset separately and the combined dataset. From this data we used information on age, race, Hispanic ethnicity, family history of ovarian or breast cancer, exercise habits, drinking habits, smoking habits, BMI, diabetes, ulcers, asthma, emphysema, stroke, hypertension, heart disease, and any previous cancers. All the factors were transformed to lay on a 0–1 scale before being used as input to the NN. For the categorical variable, race, we used one-hot encoding (Bishop, 2006). In the past we have discarded respondents with missing data (Roffman et al., 2018a,b), here we also used the idea of one-hot encoding to handle missing data. Basically, for every factor we created a variable indicating whether a respondent had provided a response. The breakdown of the data set is given in Table 1. We divided the data into two sets: training (70%) and testing (30%). This split was done randomly while keeping the ratio of cancer to non-cancer cases constant.

**Table 1:** Personal health parameters from the NHIS and PLCO datasets used in the ANN.

	**NHIS**				**PLCO**			
	**Cancer**		**No cancer**		**Cancer**		**No cancer**	
	**Mean (SD)**	**% Missing**	**Mean (SD)**	**% Missing**	**Mean (SD)**	**% Missing**	**Mean (SD)**	**% Missing**
**CONTINUOUS VARIABLES**								
Age	42.3 (17.4)	0.0	48.5 (18.5)	0.0	69.5 (6.5)	0.0	73.6 (5.9)	0.0
Diabetes age	44.1 (17.4)	7.1	48.1 (17.4)	2.5	N/A	100.0	N/A	100
Smoking age	18.4 (6.3)	0.7	19.2 (7.8)	1.5	20.3 (6.4)	1.5	19.6 (5.6)	0.4
Years quit	8.6 (12.2)	0.3	16.5 (13.9)	1.0	25.8 (13.1)	0.6	30.5 (12.5)	2.0
Pack-years	20.8 (22.1)	52.0	17.3 (17.9)	52.5	27.7 (22.2)	2.0	30.3 (25.3)	2.1
Vigorous exercise	56.9 (221.6)	3.0	66.0 (190.1)	4.3	N/A	100.0	N/A	100
Moderate exercise	108.0 (285.1)	4.1	106.9 (264.1)	5.3	N/A	100.0	N/A	100
Drinking frequency	60.4 (102.1)	0.6	63.2 (92.0)	1.2	N/A	100.0	N/A	100
Drinking amount	2.1 (1.7)	21.3	2.1 (2.0)	11.5	N/A	100.0	N/A	100
Binging frequency	6.7 (26.3)	21.8	6.4 (28.9)	12.1	N/A	100.0	N/A	100
Family members with breast cancer	0.29 (0.84)	86.5	0.23 (0.76)	85.4	1.86 (4.51)	3.9	1.72 (4.46)	3.3
Family members <50 with breast cancer	0.14 (0.61)	86.5	0.12 (0.60)	85.4	0.41 (2.08)	0.0	0.42 (2.11)	0.0
Family members with ovarian cancer	0.34 (1.13)	86.3	0.08 (0.57)	85.3	0.67 (2.74)	3.9	0.44 (2.24)	3.3
Family members <50 with ovarian cancer	0.23 (0.89)	86.3	0.05 (0.42)	85.3	0.21 (1.52)	0.0	0.13 (1.19)	0.0
BMI	28.4 (7.6)	3.7	27.1 (6.6)	5.3	27.0 (5.7)	3.5	27.1 (5.6)	4.1
**DISCRETE VARIABLES**								
Emphysema	4.7%	0.1	1.6%	0.1	0.9%	3.7	2.1%	3.3
Asthma	20.2%	0.1	12.8%	0.1	N/A	100.0	N/A	100.0
Stroke	7.7%	0.3	3.0%	0.1	2.0%	3.5	2.1%	3.3
Coronary heart disease	8.9%	0.4	3.6%	0.2	3.8%	3.9	4.9%	3.4
Angina pectoris	5.9%	0.4	2.3%	0.2	N/A	100.0	N/A	100.0
Heart attack	8.4%	0.4	2.6%	0.1	3.8%	3.9	4.9%	3.4%
Other heart disease	17.3%	0.2	8.2%	0.1	N/A	100.0	N/A	100.0
Ulcer	20.4%	0.1	8.0%	0.2	N/A	100.0	N/A	100.0
Drink	75.0%	0.7	71.2%	1.2	N/A	100.0	N/A	100.0
Other cancer	7.8%	0.0	0.0%	0.0	2.4%	0.0	0.1%	0.0
Hypertension	45.8%	0.0	30.1%	0.0	34.7%	3.3	36.2%	3.3
Hispanic	11.3%	0.0	16.2%	0.0	1.6%	4.3	1.8%	5.0
Diabetes:		0.1		0.1		3.5		3.3
Diabetic	6.0%		8.4%		4.7%		6.4%	
Prediabetic	0.0%		1.4%		N/A		N/A	
Not Diabetic	94.0%		90.2%		95.3%		93.6%	
Smoking:		0.5		0.7		0.0		0.0
Current	21.7%		18.0%		8.9%		9.5%	
Former	27.9%		18.8%		34.7%		33.7%	
Never	50.4%		63.2%		56.4%		56.8%	
Smoking frequency:		1.0		2.0		0.0		0.0
Every day	36.3%		38.9%		20.4%		21.9%	
Some day	7.4%		10.0%		N/A		N/A	
Quit	56.3%		51.1%		79.6%		78.1%	
Race:		0.0		0.0		0.0		0.0
White	82.4%		73.4%		89.4%		86.2%	
Black	10.3%		15.8%		3.5%		5.6%	
AINA	1.2%		0.9%		0.2%		0.3%	
Asian Indian	0.0%		0.7%		2.4%		3.3%	
Chinese	0.3%		0.9%		2.4%		3.3%	
Filipino	0.6%		1.0%		2.4%		3.3%	
Other	4.9%		6.8%		0.2%		0.5%	
Multiracial	0.2%		0.3%		N/A		N/A	

The NHIS monitors the overall health status of the United States. Each year roughly 30,000 people are surveyed through in-person interviews on a broad range of health topics (Centers for Disease Control and Prevention, 2017). They are asked about their current and past health, making this retrospective data. The survey has evolved over the years, so we used the data from 1997 to 2017 because of the consistency in the survey over those years. In this dataset there are 3,61,374 female respondents; 1,418 were diagnosed with ovarian cancer previous to the survey. Since a person's health can vary over time, especially if they have cancer, the data for those diagnosed may not be useful if too much time has passed between the diagnosis and the survey. Therefore, in using the NHIS data we create multiple data sets (NHIS-X) where those diagnosed within X years of taking the survey are marked as cancer positive and those diagnosed more than X years before the survey are removed from the data. We set X to one through eight which keeps ~10–40% of the cancer cases, respectively. As discussed below the lowest year cutoff was chosen for the final model.

The PLCO comes from a randomized, controlled trial investigating the effectiveness of various screenings for prostate, lung, colorectal, and ovarian cancer. Participants were enrolled from November 1993 through July 2001 and followed for 13 years (Kramer et al., 1993), making this data prospective. There were 78,215 female participants, 461 were diagnosed with ovarian over the course of the study. A comparison of the two data sources has been summarized in Table 1.

### Neural Network

As the name implies a neural network is a network of neurons or nodes, with each neuron resembling a logistic regression. A neuron's inputs, the output of the preceding layer's neurons, are combined in a weighted sum with an intercept or bias term. This sum is fed to an activation function, typically a sigmoidal function such as the logistic or tanh function, to produce the neuron's output. The simplest neural network consists of an input layer and output layer and is equivalent to a logistic regression. The input layer consists of the model's input data. The output layer's output is what the model returns to the user. Layers added between the input and output layer are known as hidden layers and get their inputs from the output of the previous layer and likewise their output becomes the input of the next layer (Bishop, 2006).

Our NN is fit using an in-house MATLAB code that takes about 2 h per model. Our NN only has one neuron in the output layer, representing the probability an individual has ovarian cancer. We evaluated models having 0 (logistic regression) to 3 hidden layers with 4–12 neurons per hidden layer (Table 2). For our activation function we used the logistic function. We used the sum of squared errors as our loss function. We used the standard backpropagation algorithm with the learning rate updated with the momentum (Bishop, 2006). The network is trained to identify which respondents have cancer. However, the raw output of the NN is a number between 0 and 1. Turning this into a binary result requires selecting a threshold above which is considered a 1 and otherwise 0. The sensitivity and specificity are calculated as a function of this threshold. The final threshold is selected to maximum the sum of the sensitivity and specificity. In addition to selecting a threshold value we will use the raw output of the network, which we refer to as the respondents' risk.

**Table 2:** Mean AUC ± one standard deviation for 10-fold cross validation testing different architectures and data combinations.

	**0 Hidden layers**	**1 Hidden layer**	**2 Hidden layers**		**3 Hidden layers**	
	1	12-1	8-8-1	12-12-1	**12-8-4-1**	12-12-4-1
PLCO	0.59 ± 0.078	0.65 ± 0.033	0.64 ± 0.040	0.66 ± 0.027	0.65 ± 0.037	0.65 ± 0.037
NHIS-1	0.58 ± 0.129	0.59 ± 0.131	0.60 ± 0.132	0.57 ± 0.129	0.56 ± 0.130	0.60 ± 0.131
NHIS-2	0.59 ± 0.109	0.60 ± 0.109	0.62 ± 0.109	0.57 ± 0.107	0.55 ± 0.108	0.60 ± 0.109
NHIS-3	0.57 ± 0.096	0.59 ± 0.096	0.58 ± 0.096	0.57 ± 0.096	0.56 ± 0.095	0.58 ± 0.096
NHIS-4	0.59 ± 0.088	0.61 ± 0.089	0.63 ± 0.088	0.59 ± 0.089	0.58 ± 0.089	0.61 ± 0.088
NHIS-5	0.60 ± 0.084	0.61 ± 0.084	0.61 ± 0.084	0.60 ± 0.084	0.57 ± 0.084	0.58 ± 0.084
NHIS-6	0.61 ± 0.080	0.62 ± 0.080	0.61 ± 0.080	0.60 ± 0.080	0.59 ± 0.080	0.60 ± 0.080
NHIS-7	0.59 ± 0.077	0.60 ± 0.077	0.60 ± 0.077	0.59 ± 0.077	0.57 ± 0.076	0.59 ± 0.077
NHIS-8	0.63 ± 0.075	0.61 ± 0.075	0.64 ± 0.075	0.62 ± 0.075	0.61 ± 0.075	0.62 ± 0.075
**PLCO+NHIS-1**	0.72 ± 0.051	0.80 ± 0.064	0.80 ± 0.064	0.80 ± 0.064	**0.80 ± 0.064**	0.80 ± 0.064
PLCO+NHIS-2	0.66 ± 0.054	0.76 ± 0.064	0.76 ± 0.063	0.75 ± 0.064	0.76 ± 0.064	0.75 ± 0.064
PLCO+NHIS-3	0.64 ± 0.053	0.72 ± 0.062	0.74 ± 0.061	0.72 ± 0.062	0.73 ± 0.062	0.72 ± 0.062
PLCO+NHIS-4	0.61 ± 0.054	0.71 ± 0.060	0.72 ± 0.060	0.71 ± 0.061	0.72 ± 0.060	0.72 ± 0.060
PLCO+NHIS-5	0.58 ± 0.054	0.71 ± 0.059	0.71 ± 0.059	0.71 ± 0.059	0.70 ± 0.059	0.70 ± 0.059
PLCO+NHIS-6	0.57 ± 0.053	0.69 ± 0.058	0.70 ± 0.058	0.69 ± 0.058	0.70 ± 0.058	0.69 ± 0.058
PLCO+NHIS-7	0.56 ± 0.052	0.68 ± 0.057	0.68 ± 0.057	0.68 ± 0.057	0.68 ± 0.057	0.68 ± 0.057
PLCO+NHIS-8	0.54 ± 0.053	0.69 ± 0.056	0.70 ± 0.056	0.69 ± 0.056	0.69 ± 0.057	0.69 ± 0.055

*In the leftmost column, the NHIS-X stands for the NHIS data with a cutoff year of X for ovarian cancer diagnosis prior to the survey. The 2nd row headers give the number of neurons in the hidden layers and output layer. The bold indicates the selected model for further study*.

## Risk Stratification

To show how the NN could be used in the clinic we also present a risk stratification scheme. Based on the respondents' risk, we split the population into three categories: low, medium, and high-risk. The boundaries between these categories are selected using the training data. The boundaries are conservatively selected so that only 1% of those without cancer were labeled as high risk and only 1% of those with cancer were labeled as low risk.

## Results

### Model Selection

**Figure 1:**
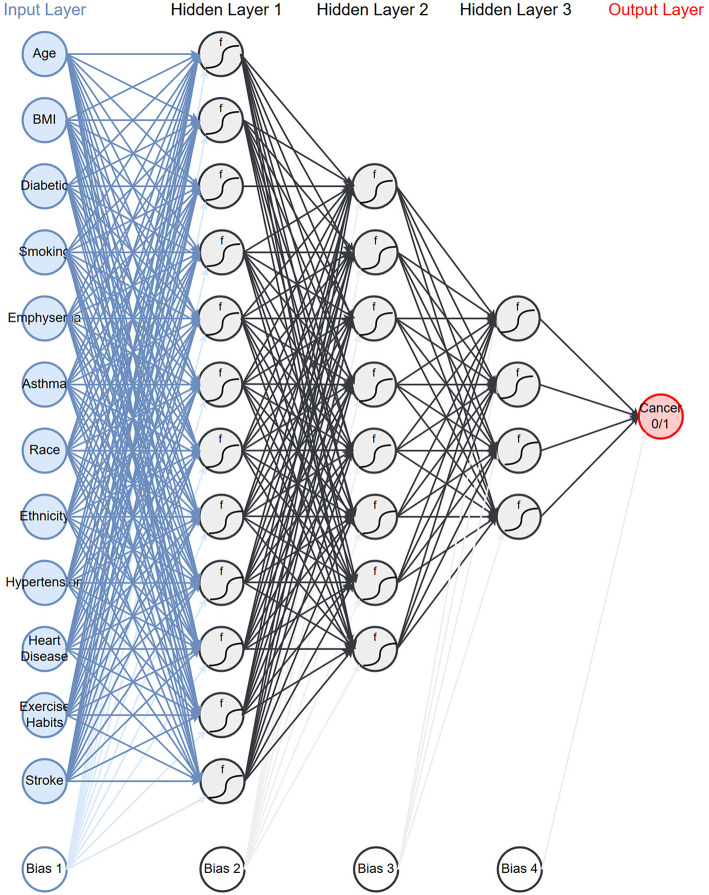
Network architecture: the neural network architecture that is chosen to use for the rest of the paper.

As described in the methods we created models on 17 different configurations of the data with six architectures. Table 2 presents the average area under the ROC curve (AUC) with one standard deviation from the 10-fold cross validation. We see that the performance ranges from an AUC of 0.54–0.80. The performance of the top models are within one standard deviation of each other and therefore not significantly different. However, for the remainder of this paper we will be focusing on a single model. We select the model which uses both PLCO and NHIS data with a 1-year lapse allowance (bold in Table 2). This model has three hidden layers with 12 neurons in first, eight in the second, and four in the last (Figure 1). This model was selected because it is tied for the highest AUC and generalizing the best, e.g., has a smaller difference in the training (data not shown), and validation AUC (Table 2) than the other models with high AUCs.

### Model Performance

Having selected a model and dataset to use, we trained the model using the full dataset and evaluated it on the testing set that was held out from the initial training. In calculating the performance of the model on both the training and testing datasets we calculated 95% confidence intervals based on the number of respondents with ovarian cancer (Hanley and McNeil, 1982).

**Figure 2:**
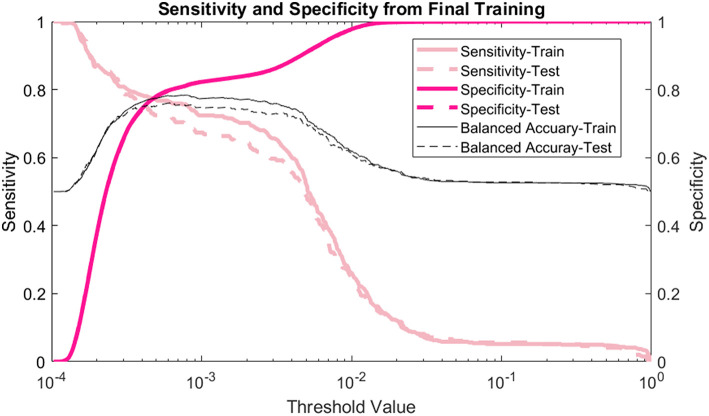
Sensitivity and specificity: the sensitivity, specificity, and balanced accuracy as a function of decision threshold.

**Figure 3:**
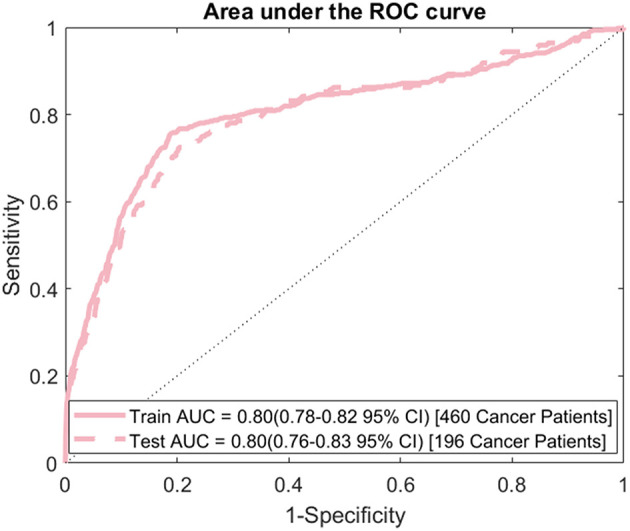
ROC curve: area under the ROC curve (AUC) for the final model evaluated on both the training and testing data.

The model's sensitivity on the training dataset was 75.7% (95% CI: 79.6–71.7%) and on the testing data it was 69.4% (95% CI: 75.8–62.9%) (Figure 2). The model's specificity was 81.3% (95% CI: 81.4–81.1%) on training and 81.2% (95% CI: 81.4–81.0%) on testing. The positive predictive value (PPV) was 0.554% (95% CI: 0.587–0.521%) for training and 0.506% (95% CI: 0.559–0.454%) for testing. The negative predictive value (NPV) was 99.96% (95% CI: 99.97–99.95%) for training and 99.95% (95% CI: 99.96–99.94%) for testing. Lastly, the AUC of the model (Figure 3) is 0.80 (95% CI: 0.78–0.82) for training and 0.80 (95% CI: 0.76–0.83) for testing, respectively.

**Figure 4:**
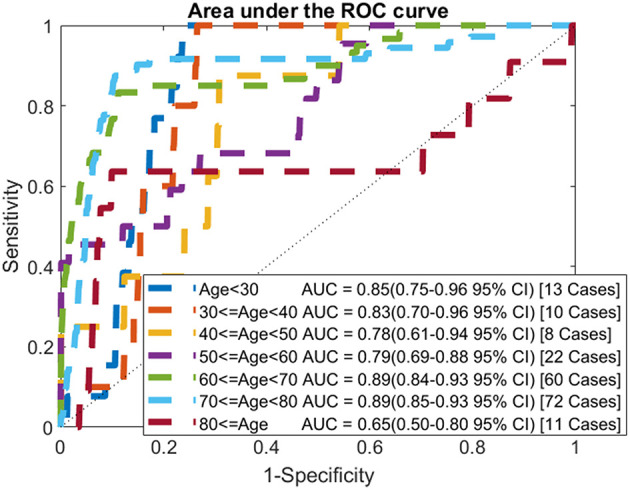
ROC curves by age: comparison of AUC values when applying the model to each age group of the testing data.

In addition to looking at the overall performance of the model, we also evaluated it for different age groups. The AUC for each age group of the testing data is shown in Figure 4. For most age groups the AUC is higher than the AUC for the model evaluated on the whole population.

### Risk Stratification

**Figure 5:**
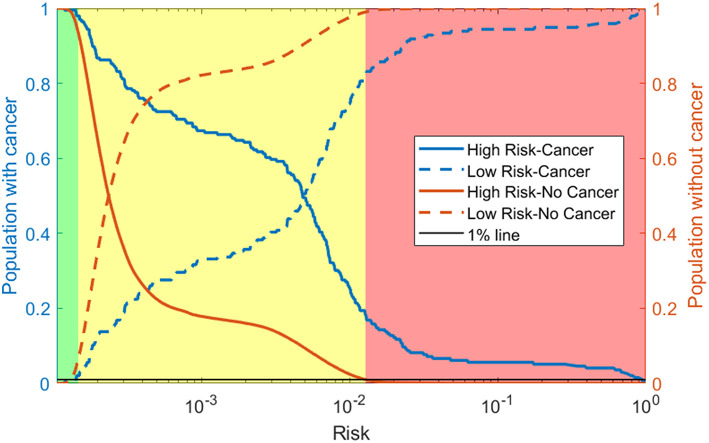
Risk stratification: cumulative distribution functions (CDFs) of the percentage of the population with cancer (blue lines) and without cancer (orange lines) classified as high risk (solid line) and low risk (dashed line) as the risk boundaries vary. We selected the boundaries based on where the 1% (black line) intersects the high-risk CDF for those without cancer and the low risk CDF for those with cancer. This gives the high (red), medium (yellow), and low (green) risk regions.

As discussed in the methods we use our model to create a 3-tiered risk stratification scheme. The risk boundaries were conservatively chosen with <1% mis-classification error. Figure 5 shows what risk values form the boundaries and three shades of red, yellow, and green representing the high, medium, and low risk, respectively. This figure also includes the cumulative distribution functions for those with and without cancer being marked as high or low risk as the risk boundaries move. This allows for more possible boundary considerations. For example, allowing 15% of those without cancer to be classified as high risk would increase the percentage of those with cancer being classified as high risk from about 20–60%.

Using our conservative boundaries, we applied this stratification scheme to the testing dataset. As can be seen in Table 3, 18.4% of those with cancer are classified as high risk and 6.4% of those without cancer are classified as low risk. The bulk of the population (~80% of those with cancer and ~90% of those without it) is classified as medium risk. This makes the prevalence of ovarian cancer 48 per 100,000, 128 per 100,00, and 2,645 per 100,000 in the low, medium, and high-risk groups, respectively.

**Table 3:** Results of the 3-tiered stratification scheme when applied to the testing dataset.

	**Low risk**		**Medium risk**		**High risk**	
	**Number**	**%**	**Number**	**%**	**Number**	**%**
Cancer cases	4	2.0	156	79.6	36	18.4
Non-cancer cases	8,346	6.4	121,400	92.6	1,325	1.0

## Discussion

We created a neural network model for ovarian cancer risk. Using readily available personal health data our model can discriminate between those with and without cancer. By stratifying the population into three risk categories, we believe our model can identify a high-risk population for whom screening would be beneficial. To avoid common pitfalls in machine learning we tested a variety of models with different data configurations using two different data sources (NHIS and PLCO).

We trained the model on the PLCO data alone, the NHIS data alone with different cutoff years and the combination of PLCO and NHIS data. The PLCO data by itself did not perform well. It is understandable because there are far fewer respondents in the PLCO data than the NHIS data. Also, the cohort in this data is older (50–75 years old) making discriminating between those with and without cancer harder as most cancer incidences happen in older women. The NHIS data by itself performed even worse. However, as the cutoff was increased the model performance was improving. This difference in performance is probably due to the number of cancer cases in the data. The longest cutoff gives about 4 times the number of cancer cases as the shortest. With few cancer cases the models with a low cutoff are likely to “memorize” the training cases and not generalize well to the validation data. The combined data outperformed either set by itself with the smaller cutoff years, for NHIS data, doing the best.

We also tested using different numbers of hidden layers and different numbers of neurons per layer. Of special note is the model with no hidden layers, which is equivalent to a logistic regression. For all the data configurations the logistic regression had the lowest AUC and for most of the configurations it is significantly lower. This indicates that the interactions of the input factors with each other is important and thus justifies the use of a neural network.

After comparing a variety of models, we selected one to further develop and use for our stratification scheme. The AUC for many of the top models were within a standard deviation of each other and therefore not significantly different. In choosing among these we considered generalization (difference in training and testing AUC) in addition to the testing AUC value. This led us to the model using the PLCO data and the NHIS data with the shortest cutoff with three hidden layers with 12 neurons in the first, eight in the second, and four in the last hidden layer.

The selected model was evaluated on the testing dataset. Comparing the performance of the model on the training and testing datasets, it is shown that the model generalizes well with all measures of performance being similar. We also calculated the AUC for each age group. While the number of cancers in each group is small we find that for most groups the AUC is higher than the AUC when evaluated on the whole population. Unsurprisingly, for the younger age groups, where ovarian cancer is uncommon, the AUCs are very high (0.85 for under 30 and 0.83 for those in their 30s). Most importantly the model performs well for the older age groups with the AUC for those in their 50s comparable to the model's AUC on the whole population and the AUC for those in their 60s, and 70s being significantly higher than the whole model's AUC.

Comparing to the risk prediction model of Pfeiffer et al. (2013) our model performs significantly better (0.59 vs. 0.80 AUC). This is particularly interesting because they also used PLCO as one of their two datasets. Additionally, they used data known to be important to ovarian cancer, such as hormone therapy and menopausal status data. The QCancer® algorithm is the other model that predicts ovarian cancer risk with readily available personal health data. Compared with QCancer® our model is not as strong (0.86 vs. 0.80 AUC) (Collins and Altman, 2013). QCancer® has two advantages over our model. First, it was trained with an order of magnitude more data. Also, they included ovarian cancer specific symptoms, such as post-menopausal bleeding, which were unavailable to us. Nevertheless, our model has performed well and is highly discriminatory between those with and without ovarian cancer, specifically for older women. With this great performance it is likely our model would do even better and possibly outperform QCancer® if we had more features known to affect ovarian cancer risk such as birth control use, hormone therapy, or menopausal status.

Whereas general screening of older women for ovarian cancer has more harms than benefits (Jacobs et al., 1999, 2015; Buys et al., 2011; Grossman et al., 2018), we believe that our model's discriminatory power can be used to refine the population who receives regular screening and hopefully tip the harms/benefit ratio. With a PPV of 0.506%, the population marked positive by our model has a prevalence of ovarian cancer of 506 per 100,000 people which is almost a 4-fold increase over the prevalence in the whole population, 138 per 1,00,000 (SEER: US Population Data 1969-2015 with Other Software, 2016). However, the strength of our model lies in stratifying the population by risk. With our conservative risk boundaries, the model classified almost 20% of those with cancer as high risk making the prevalence in this category (2,645 per 100,000) 20 times higher than that of the whole population. This is comparable to high risk populations identified through genetic testing; the lifetime risk for ovarian cancer increases 27 and 11 times with BRCA1 and BRCA2 mutations, respectively (Kuchenbaecker et al., 2017). Note that our neural network achieves this 20-fold increase in discriminatory power for high-risk group based solely on personal health data, without any genomic testing, nor transvaginal ultrasound, or CA-125 serum testing. Accordingly, we suggest regular screenings for our high-risk group would likely provide more benefits than harms. Our model classified more than 6% of those without cancer as low risk. We suggest that this group may not need to be screened at all. Finally, our model classified about 80% of those with and 90% of those without cancer as medium risk. We suggest that this population might benefit from infrequent screening and perhaps monitoring on a positive screening instead of, potentially harmful, intervention. As mentioned in the results, depending on the economic cost and harms/benefit trade-off, less conservative boundaries could be selected, resulting in more people with cancer classified as high risk.

## Conclusion

We present a neural network that uses readily available clinical data to stratify the population in terms of ovarian cancer risk. Using this data makes the model cost-effective and non-invasive compared to traditional screening modalities. While screening the general population may have no net benefit, this model could help identify high risk groups who would benefit from tailored screening.

## Author Contributions

GRH analyzed data, produced results, and wrote technical details. BN, WM, and YL provided technical consultation and reviewed the manuscript. GSH provided clinical consultation and reviewed the manuscript. JD generated research ideas and reviewed the manuscript.

### Conflict of Interest Statement

The authors declare that the research was conducted in the absence of any commercial or financial relationships that could be construed as a potential conflict of interest.
